# Cryo-EM and directed evolution reveal how *Arabidopsis* nitrilase specificity is influenced by its quaternary structure

**DOI:** 10.1038/s42003-019-0505-4

**Published:** 2019-07-17

**Authors:** Andani E. Mulelu, Angela M. Kirykowicz, Jeremy D. Woodward

**Affiliations:** 1 Division of Medical Biochemistry and Structural Biology, Department of Integrative Biomedical Sciences, University of Cape Town, Anzio Road, Observatory, Cape Town, 7925 South Africa; 2 Structural Biology Research Unit, University of Cape Town, Cape Town, 7925 South Africa

**Keywords:** Hydrolases, Protein design, Secondary metabolism, Biocatalysis, Electron microscopy

## Abstract

Nitrilases are helical enzymes that convert nitriles to acids and/or amides. All plants have a nitrilase 4 homolog specific for ß-cyanoalanine, while in some plants neofunctionalization has produced nitrilases with altered specificity. Plant nitrilase substrate size and specificity correlate with helical twist, but molecular details of this relationship are lacking. Here we determine, to our knowledge, the first close-to-atomic resolution (3.4 Å) cryo-EM structure of an active helical nitrilase, the nitrilase 4 from *Arabidopsis thaliana*. We apply site-saturation mutagenesis directed evolution to three residues (R95, S224, and L169) and generate a mutant with an altered helical twist that accepts substrates not catalyzed by known plant nitrilases. We reveal that a loop between α2 and α3 limits the length of the binding pocket and propose that it shifts position as a function of helical twist. These insights will allow us to start designing nitrilases for chemoenzymatic synthesis.

## Abstract (web summary)

Andani Mulelu et al. solve the structure of *Arabidopsis* nitrilase NIT4 using cryo-EM at a 3.4 Å resolution. They show that mutation of three residues alters substrate specificity due to a substantially rearranged supramolecular architecture.

## Introduction

Branch 1 nitrilases (EC 3.5.5.1) (NITs) are industrially important^1^ oligomeric enzymes (forming left-handed twists, spirals, turns or helices) that share a common architecture^2–8^ and catalyze the hydrolysis of nitriles to carboxylic acids and ammonia and/or amides^9^. In all plants, the nitrilase 4 (EC 4.2.1.65) (NIT4) group represents the ancestral form, which is highly specific for β-cyano-l-alanine^10^. The derived NIT1 group, which is restricted to the *Brassicaceae* family includes NIT1, NIT2, and NIT3, is believed to have arisen from multiple gene duplication events^11^. NIT1s generally display measurable activity against a large range of aliphatic and aromatic nitriles, but cannot catalyze the hydrolysis of β-cyano-l-alanine^12^, while NIT4 shows either extremely minor (<0.75%) or no activity for the best substrates of the NIT1 group^10^. It is thought that the NIT1 group enzymes evolved to catabolise the diverse glucosinolate-derived nitriles found in the *Brassicaceae*^11–13^. Interestingly, plant NITs with high specificity against a single substrate or narrow group of substrates have evolved from the ancestral generalist NIT1 group enzymes^8,13,14^.

We recently discovered that substrate specificity in plant NITs is related to the structure of the NIT helical assembly^8^. The helical twist (Δ*ϕ*) of the NIT filament is correlated with the size of the preferred substrate: NIT4 enzymes have a relatively large helical twist, small substrate size, and high specificity, while NIT1 enzymes have a smaller twist and broader specificity. Specialized NIT1 enzymes that have evolved higher specificity and small substrate size show a larger helical twist^8^ . Helical twist is not the only determinant of specificity: we have identified plant NITs with similar twists, but different substrate specificities. In this case, exchanging amino acid residues lining the proposed active site pocket led to a change in substrate preference, indicating that these regions play a role in substrate recognition^8^. The molecular details of this mechanism still remain speculative because only a low-twist helical NIT has been visualized crystallographically: the NIT from *Synechocystis sp*. PCC6803 (BAA10717.1) (Nit6803) ∆291 (pdb id: 3wuy^15^) (Δ*φ* = −60°).

**Fig. 1:**
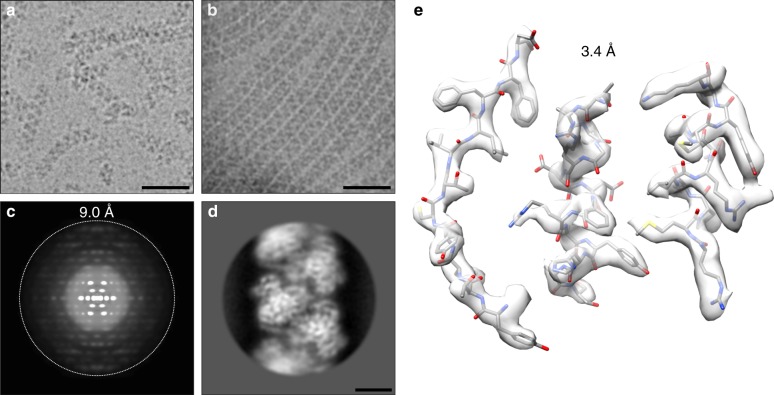
Data quality. **a** In our hands purified recombinant *At*NIT4 forms relatively short heterogeneous filaments (scale bar = 40 nm). **b** The sample after incubating and blotting in a low-humidity environment: the concentrating effect of dehydration resulted in the formation of a monolayer of long, straight filaments stacked up against one another (scale bar = 40 nm). **c** The filaments showed helical order to a limit of ~9 Å. **d** Clear secondary structure can be visualized in the classified average, but this deteriorates with increasing distance from the center because of the curved nature of the filaments (scale bar = 5 nm). **e** By masking out a single helical turn and treating this as a single particle we were able to improve the resolution substantially

Here we present what we believe to be the first close-to-atomic structure of a full-length active helical NIT (EC 3.5.5.1) using cryo-electron microscopy at a resolution of 3.4 Å. We compare the structures of *At*NIT4 (with a large twist, high specificity and small substrate) to Nit6803^15,16^ (with small twist, broad specificity and large substrates). We exploit the insights gained in a targeted directed evolution approach^17,18^ to generate a NIT4 mutant with high activity against substrates not converted by either NIT4 or NIT1 and which shows no activity against the substrate of NIT4. As predicted by our previous work^8^, this enzyme shows a large decrease in helical twist.

## Results

### The structure of *At*NIT4

To study the molecular details of helical twist on substrate specificity we reconstructed an active plant NIT by high-resolution cryo-electron microscopy. Preliminary screening of plant NITs revealed a general pattern of heterogeneity with limited helical order. In general, NIT4 produced better filaments than NIT1, but these were still somewhat curved, short and broken (Fig. 1a). We screened buffer conditions and protein concentration in an attempt to improve filament quality without success. Eventually, we unexpectedly discovered that by incubating and blotting freshly prepared *Arabidopsis thaliana* nitrilase 4 (*At*NIT4) (NP_197622.1) before vitrification in a 10% humidity, 25 °C environment, we could improve the quality of the imaged fibers (Fig. 1b). Despite this, we only observed reflections to a resolution of ~9 Å after vertical alignment (Fig. 1c). Clear secondary structure was visible in classified averages, but with a rapid loss of resolution with increasing distance from the center of the images, indicating poor long-range helical order (Fig. 1d). Treating one helical turn (five dimers) as a single particle and selecting a homogeneous dataset through rounds of 2D and 3D classification allowed us to achieve a resolution of 3.4 Å (gold-standard Fourier shell coefficient (FSC) = 0.143) (EMD-0320, pdb id: 6i00) (Fig. 1e and Table 1).

**Table 1:** Cryo-EM data collection, refinement, and validation statistics. *At*NIT4: EMD-0320, pdb id: 6i00

	*At*NIT4 EMD-0320 PDB 6i00
Data collection	
Microscope	Titan Krios
Voltage (kV)	300
Pixel size (Å)	0.85
Defocus range (µm)	−0.75 to −2.0
Exposure time (s)	7
Dose rate (e^−^ pixel^−1^ s^−1^)	6.5
Number of images	1266
Number of frames per image	45
Map reconstruction	
Initial particle number	392,263
Final particle number	133,106
Box size (pixels)	260
Helical twist (°)	−72.98
Helical rise (Å)	17.48
Number of monomers per box	25.3
Box overlap (%)	85
Resolution (Gold-standard FSC = 0.143) (Å)	3.4
Map sharpening *B* factor (Å^2^)	−124
Model composition	
Non-hydrogen atoms (monomer)	2262
Protein residues (all/built)	355/289
Ligands	0
Fit to map	
Correlation coefficient (entire box)	0.759
Correlation coefficient (around atoms)	0.823
FSC = 0.5 (between model and map) (Å)	3.49
Protein geometry	
Molprobity score	1.69
All-atom clash score	2.29
EMRinger score	3.82
RMSD bonds (Å)	0.006
RMSD angles (°)	1.153
Ramachandran favored (%)	83.92
Ramachandran allowed (%)	15.30
Ramachandran disallowed (%)	0.78

### Helical structure of NIT4

**Fig. 2:**
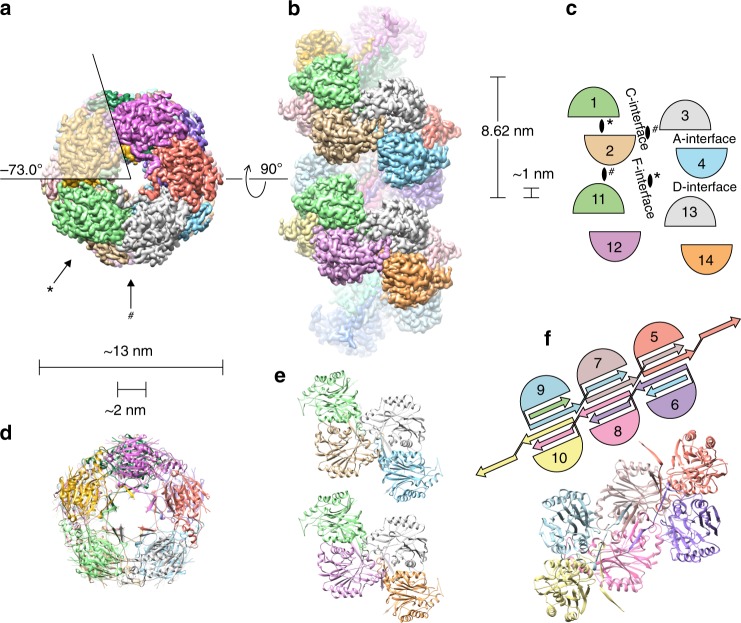
NIT helical structure. **a** In vitro*, At*NIT4 assembles into an extended left-handed helical tube with 4.9 subunits per turn (helical twist of −73.0°) and outer and inner diameters of ~13 nm and ~2 nm, respectively. Arrows indicate the positions of the two diad-axes (#) and (*). **b**, **c** The NIT4 filament is comprised of a left-handed helix with a pitch of 8.62 nm. Monomers associate across previously described interfaces^2,4^; while no interaction was observed across the D- or F-interfaces, they are shown for the sake of completeness. The positions of the two diad-axes (#) and (*) relative to individual monomers are shown (EMD-0320). **d** About 80% of the model was built with high certainty, 34 N- and 8 C-terminus residues and 24 residues on the outer surface of the filament were disordered and could not be visualized in the map. ß-sheets stabilizing the helical structure can be seen in the core of the enzyme (pdb id: 6i00). **e** The C-interface is predominantly an interaction across monomers 2 and 3. **f** The C-terminal tail of each sequential monomer contributes two ß-strands, which form one ß-sheet for every dimer

*At*NIT4 is a left-handed helical tube with 4.9 sunbunits (dimers) in one helical turn, a pitch of 8.62 nm, an outer diameter of ~13 nm, and a ~2 nm hollow core (Fig. 2a, b). There are two diad axes, which are related to one another by a −5° (negative value: left-handed by convention) rotation and 4.31 nm translation along the helical axis. The first passes through the C- and D-interfaces^2^ and the second passes between the A- and F-interfaces^2,4^ (Fig. 2c). A ~1 nm gap (Fig. 2b) across the helical groove shows that contrary to what has been observed previously in negative stain^8^, this region lacks stabilizing interactions (D- or F-interfaces). Amino acid residues involved in the formation of helical interfaces are shown in Supplementary Fig. 1. Interestingly, the predominant interaction stabilizing the filament is between the C-terminal tails of four adjacent monomers, which all contribute strands that form a row of ß-sheets in the core of the enzyme (Fig. 2d, f).

### The substrate-binding pocket

**Fig. 3:**
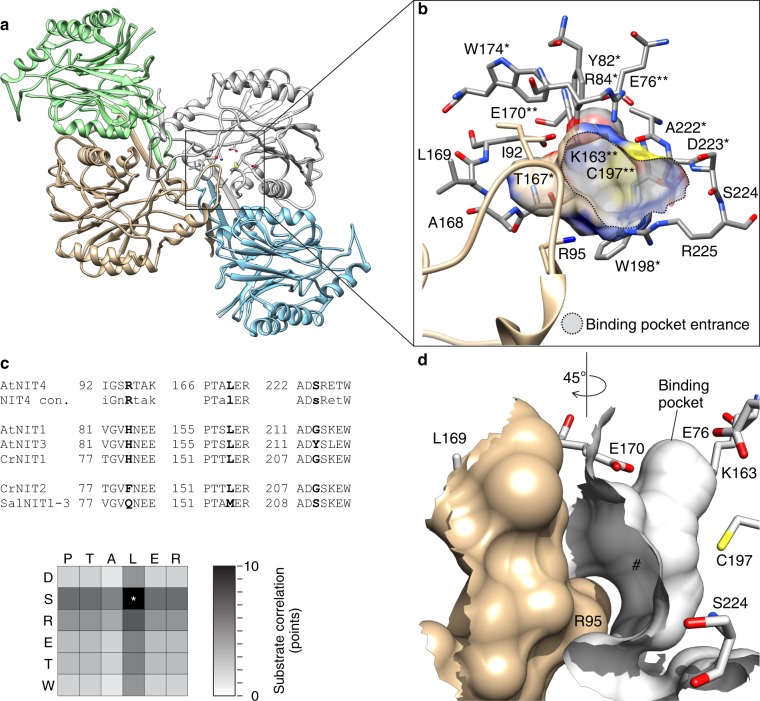
The substrate-binding pocket. **a** *At*NIT4 structure showing two dimers interacting across the C-interface, the position of the catalytic site is indicated by a square. **b** The active-site pocket, the catalytic residues (E76, K163, E170, and C197) are shown (**), residues conserved in all plant NITs (*). A loop (tan) arising from the adjacent monomer (between α2 and α3) extends over the entrance to the active-site pocket. **c** As expected, the amino acid residues surrounding the binding pocket correlate with substrate specificity. Three amino acid positions in particular are responsible for the majority of the variation between known plant NITs: 169, 224, and 95 (numbered according to *At*NIT4). **d** A lid (tan), formed by the loop, limits the length of the binding pocket (#). R95 is in the lid loop; S224 is on the outer border of the binding pocket and L169 points toward the light green subunit across the C-interface

*At*NIT4 shows high-structural homology with other members of the NIT superfamily including a conserved αßßα core (Fig. 3a) and CEEK catalytic tetrad (Fig. 3b and Supplementary Fig. 2)^19,20^. The substrate-binding pocket is a ~7 × 4 Å tunnel lying close to the C-interface (Fig. 3a) that runs almost perpendicular to the helical axis (Fig. 3b). NITs have an insertion between α2 and α3 relative to crystallized NIT superfamily members^21^, in Nit6803 ∆291 this loop forms a twofold interaction across the C-interface distant from the active-site pocket (pdb id: 3wuy^15^). Plant NITs have a further five-residue insertion relative to Nit6803 (Supplementary Fig. 1), which conveys this loop across the C-interface, forming a lid over the symmetry-related substrate-binding site (Fig. 3a, b, d).

### The mode of substrate binding

There is good biochemical evidence to suggest that the substrate (or a substrate intermediate) binds covalently to the NIT active-site cysteine^10,22^. This requires that the nitrile group of the substrate be orientated correctly relative to C197 and imposes a strong constraint on the possible position of the bound substrate. We produced a structural alignment between *At*NIT4 and the structures of three NIT superfamily enzymes cocrystallized with substrates/intermediates. The structures used were: a C171A/V236A mutant of N-carbamyl-D-amino acid amidohydrolase from *Agrobacterium sp*. KNK712 complexed with N-carbamyl-D-methionine (pdb id: 1uf5)^23^; an amidase from *Pseudomonas aeruginosa* with trapped acyl transfer intermediate (pdb id: 2uxy)^24^ and a C145A mutant of the amidase from *Nesterenkonia* AN1 complexed with butyramide substrate (pdb id: 4izs)^25^. The substrates/intermediates localize to the binding pocket and lie in the direction of the lid loop. Their fit is suboptimal though, and the ends of the substrates clash with binding pocket residues on one side, including the lid loop in the case of the rather long substrate N-carbamyl-D-methionine visualized in 1uf5^23^ (Supplementary Fig. 2).

### Identifying determinants of specificity

Eleven of the seventeen amino acid residues lining the binding pocket are conserved among all plant NITs regardless of substrate preference (Fig. 3b), many are conserved across all NITs. Of the four loops making up the boundaries of the binding pocket (Supplementary Fig. 1), three show sequence differences between different plant NITs (Fig. 3c). We previously exchanged residues in the (PTSLER) and (ADGSKEW) motifs in a NIT1 and observed changes in substrate preference, while exchanging a single residue in the NIT1 lid loop (VGV**H**NEE) altered helical twist, substrate size, and degree of specificity^8^. This residue is a conserved arginine in all known NIT4 enzymes that accept β-cyano-l-alanine as a substrate (EC 4.2.1.65), NIT1s have a histidine in this position and specialized NIT1s show various residues, which are correlated to their specificity and helical twists^8^. We further refined this analysis to isolate the impact of substrate specificity of individual positions within each of these motifs among the known plant NITs and identified three residue positions with a disproportionate effect on specificity (Fig. 3c and Supplementary Table 1).

### We randomized the three residues to alter specificity

**Fig. 4:**
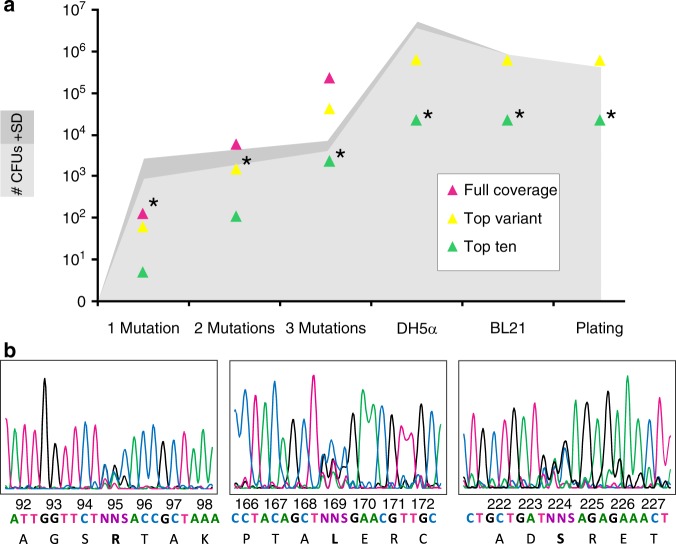
Generating the library. Mutations were introduced sequentially: R95 (1 mutation), R95 + L169 (2 mutations) and R95 + L169 + S224 (3 mutations). **a** Graphical representation of the bottlenecks in the experiment. As the library was assembled (one mutation to three mutations), more colony forming units (CFUs) are required to satisfactorily sample the full library diversity. Triangles represent the number of unique mutants present required for 95% probability of full-coverage (all possible protein mutants present), top-mutant (the best amino acid sequence present) and top-ten (one of the 10 best amino acid sequences present) in magenta, yellow, and green, respectively^26^. The upper (dark gray) and lower (light grey) 95% confidence interval values for number of CFUs show that by the end of library creation at least one of the top ten mutants was present (stars). This diversity was maintained through all cloning and selection steps. **b** The final library mix was sequenced and showed successful overlapping NNS peaks at amino acid positions 95, 169, and 224 (sample size: 1% of the total CFUs in each sample)

We performed a directed evolution experiment to identify mutants with altered substrate specificity. Target sites (R95, L169, and S224) were randomized (Supplementary Table 2), resulting in a library containing ~5.0 × 10^3^ unique mutants, sufficient to sample at least one of the top ten possible mutants with 95% probability^26^ (Fig. 4a and Supplementary Table 3). We implemented a strategy of site-saturation mutagenesis of all three sites before selection in order to avoid missing out on synergistic effects between the sites^27^, which could confound a sequential mutagenesis strategy. To maintain the full diversity of the mutant library in the selection step, 4 × 10^6^ DH5α and 7.7 × 10^5^ BL21 cells were transformed and 4 × 10^5^ cells were plated out to ensure that each unique mutant was present (Fig. 4a). The final library was sequenced as a mix (Fig. 4b) and individual colonies (Table 2) to monitor the successful incorporation of mutations.

**Table 2:** Colonies were sequenced at every stage. No bias toward wild-type sequence or multiple transformants/mixed colonies was observed

		Colony 1	Colony 2	Colony 3
1 Mutation	R95	GAC (Asp)	CGG (Arg)	TAC (Tyr)
	L169	CTT (Leu)	CTT (Leu)	CTT (Leu)
	S224	TCA (Ser)	TCA (Ser)	TCA (Ser)
2 Mutations	R95	GAC (Asp)	TCC (Ser)	GCC (Ala)
	L169	TCG (Ser)	CGC (Arg)	ACC (Thr)
	S224	TCA (Ser)	TCA (Ser)	TCA (Ser)
3 Mutations	R95	TAC (Tyr)	GGC (Gly)	CCC (Pro)
	L169	GCG (Arg)	CGC (Arg)	AAC (Thr)
	S224	CGG (Arg)	CCC (Pro)	GAC (Asp)

### We identified a NIT4 mutant with broad substrate specificity

**Fig. 5:**
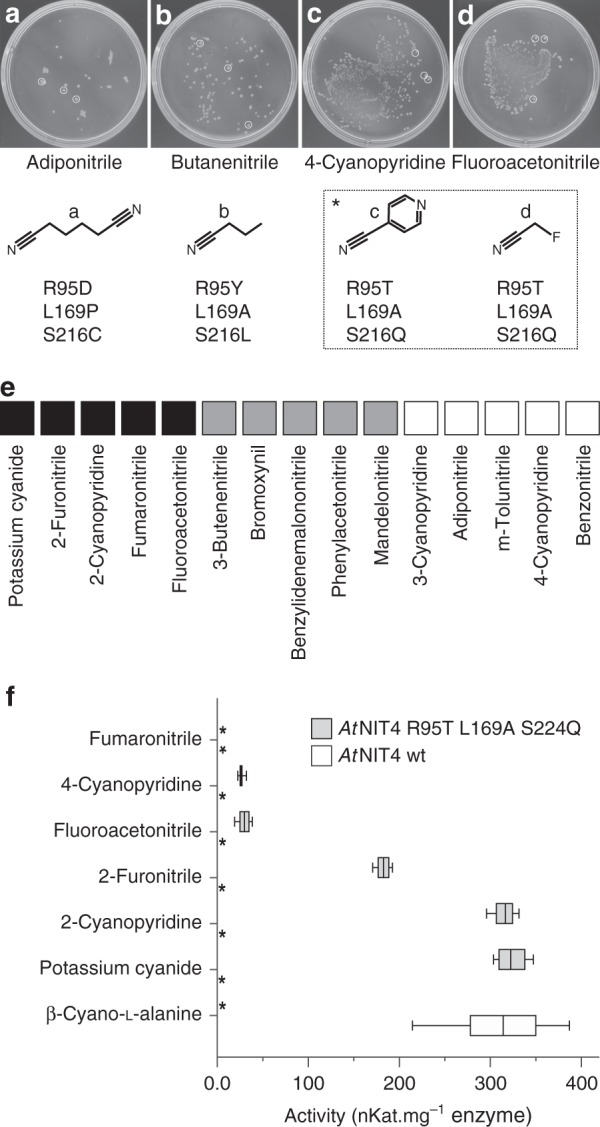
NIT4 mutant selection, screening and characterization. **a**, **b**, **c**, **d** A variable number of colonies were visible after 2 days of incubation on the selection plates. The plates, substrate structures, and sequencing results are shown. **e** Screening results indicate measured absorbance after incubating with *At*NIT4 wt, *At*NIT4 R95T L169A S224Q and an inactivated enzyme control, and assaying with Nessler’s reagent. Black indicates that *At*NIT4 R95T L169A S224Q produced more ammonia than *At*NIT4 wt. Gray indicates that the substrate reacts to form a high-absorbance product, but after incubating with the mutant, the absorbance was reduced. White indicates a slightly higher *At*NIT4 wt activity compared to the mutant. **f** Enzyme activity results, asterisk indicates no measurable ammonia formation after incubating for 30 min with 5× excess enzyme. *At*NIT4 wt showed no nitrilase activity against any of the listed substrates except for β-cyano-l-alanine. The mutant showed high activity against potassium cyanide, 2-cyanopyridine and 2-furonitrile and moderate activity against fluoroacetonitrile and 4-cyanopyridine (1st quartile, median and 2nd quartile ± sd) *n* = 3 independent activity measurements

In order to select *At*NIT4 mutants capable of hydrolyzing a specific nitrile, BL21 *Escherichia coli* cells harboring mutant enzymes were plated onto minimal media plates containing Isopropyl β-D-1-thiogalactopyranoside (IPTG) and one of 42 different nitriles (Supplementary Table 4). The basis for this assay is (1) nitrile toxicity and (2) the fact that nitrile hydrolysis produces ammonium, which can be used as a nitrogen source by the cell^28^. Cells expressing NITs capable of hydrolyzing the nitrile present in each plate will therefore have a competitive advantage, form a colony and be identified. After incubation for 13 days, between 1 and 300 colonies were observed on 15 of the nitrile plates (Supplementary Table 1). Growth on the adiponitrile, butanenitrile, 4-cyanopyridine, fluoroacetonitrile plates occurred after 2 days (Fig. 5a–d). Wild-type colonies were observed on the positive-control plate (β-cyano-l-alanine) but not on negative-control plates. Three colonies were randomly picked for sequencing from the most rapidly growing isolated colonies (Fig. 5a–d).

At least three different sequences were present on the adiponitrile plate and these were generally mixed, showing that multiple mutants had the capacity to hydrolyze this substrate. Further investigation revealed that very small but visible colonies were present on negative-control adiponitrile selection plates. Purified *At*NIT4 enzyme also showed low but measurable activity against adiponitrile (Fig. 5e). These mutants were therefore excluded from further study. All of the colonies picked from the butanenitrile plate showed the same sequence, but attempts to purify this enzyme were unsuccessful and there was no evidence of helical filaments in any of the purification fractions. Interestingly, all three of the colonies from each of the 4-cyanopyridine and fluoroacetonitrile plates gave the same sequence. Since this indicated that the mutant had the potential for broad substrate specificity, we purified this enzyme and performed a substrate screen against our set of 43 nitriles (Supplementary Table 4). This led to the identification of a further four potential substrates: fumaronitrile, potassium cyanide, 2-furonitrile, and 2-cyanopyridine (Fig. 5e). While an excess of purified wt *At*NIT4 showed no activity after 30 min incubation with the identified substrates, the mutant showed substantially different substrate specificity with no reduction in activity (Fig. 5f and Supplementary Table 5). We performed activity measurements in triplicate and ensured that at least four data points were observed while the reaction was in the linear range.

### *At*NIT4 R95T L169A S224Q has a reduced helical twist

**Fig. 6:**
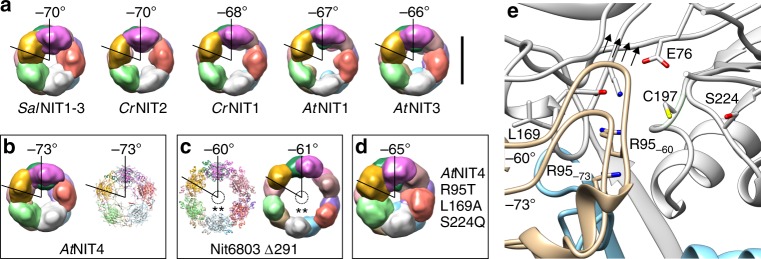
Comparison of plant NIT helical twists. **a** A comparison of different plant NITs; each monomer in the filament has been colored differently to illustrate the number of protein subunits in a helical turn (360°), the brown subunit (dimer 6) is more visible with decreasing helical twist. **b** *At*NIT4 negative stain map vs cryo-EM model, *At*NIT4 has ~5 dimers per turn. **c** Nit6803 ∆291 negative stain map vs crystal structure (**) indicates the missing C-terminal residues. **d** *At*NIT4 R95T L169A S224Q negative stain reconstruction, the low helical twist and large diameter show similarities to Nit6803 ∆291, density accounted for by the interacting C-terminal tails can be seen in the enzyme core. **e** Comparison between the structure of *At*NIT4 (labeled −73°) and *At*NIT4 with the helical operators of the Nit6803 ∆291 crystal structure applied (labeled −60°). The lid loop shifts in the direction shown by arrows (in the plane of the page), resulting in steric clashes. Geometrically, this movement is greater when the diameter of the fiber remains constant, but the helical twist decreases. Scale bar = 10 nm (applies to all end-on views)

Different plant NITs show a range of helical twists (Fig. 6a): on either side of the two extremes are NITs for which atomic models are available, *At*NIT4 (∆*φ* = −73°) and Nit6803 ∆291, which crystallized in spacegroup P6(5)^15^ (pdb id: 3wuy), meaning that it has a left-handed helical arrangement with six subunits (dimers) per turn (Δ*φ* = −60°) (Fig. 6b, c). Interestingly, while the diameter of the filament is generally ~constant between the various plant NITs, in Nit6803 ∆291 it is substantially larger. We reconstructed Nit6803 ∆291 by negative-stain electron microscopy (EMD-4407) and found that this was not a crystallization artifact and that the EM and crystal structures largely agree (Fig. 6c). We have previously observed a correlation between helical twist and the length of the preferred substrate as well as the degree of substrate specificity of a given plant NIT^8^. We therefore hypothesized that *At*NIT4 R95T L169A S224Q would show a decrease in helical twist because of its broader substrate range compared to *At*NIT4. We reconstructed *At*NIT4 R95T L169A S224Q in three dimensions by negative stain electron microscopy (EMD-4406) and found that the helical twist had changed from −73° to −65° (Fig. 6d). Unexpectedly, we also observed that the diameter of this mutant is approximately equal to that of Nit6803 ∆291 (Fig. 6c, d). Note the hollow core lacks density corresponding to the C-terminal tail (Fig. 6c).

### The effect of increasing helical twist

We compared the helical structure of Nit6803 ∆291 with that of *At*NIT4 in order to visualize the effect of the differences in helical arrangement between the two filaments. Most notably, in the Nit6803 ∆291 conformation, there is an outward rotation of ~15° about the A- and C-interfaces and a ~3 Å shift in ∆z relative to *At*NIT4. Contrary to what we proposed previously^8^, we observe an overall increase in compression of the monomer with a smaller absolute value of the helical twist. In fact, α1, α2*, α3, α4, α5, and α6 (Supplementary Fig. 1), all lying at the periphery of the enzyme are found closer to the binding pocket in Nit6803 ∆291 than in *At*NIT4.

### The role of the lid loop

The lid loop limits the length of a substrate bound to C197 (Fig. 3a, b, d). Molecular dynamics in UCSF Chimera^29^ indicates that it is held in position primarily via a charge–charge interaction between R95 and D317. In silico simulation of R95T allows the lid loop to open outward away from C197 increasing the size of the binding pocket. This explains why mutating R95 affects substrate size and specificity and provides a clue as to why this is correlated with a decrease in helical twist. Applying the symmetry operators of Nit6803 ∆291 to the structure of *At*NIT4 (loop in the closed position) results in severe steric clashes (Fig. 6e), moving the loop to the open conformation prevents this clash. This suggests that low helical twist can be used as a sign that the lid loop is in the open conformation. Interestingly, while *At*NIT4 L169A S224Q expressed insolubly, *At*NIT4 R95T (EMD-4804) showed no change in helical twist compared to the wild type. It also lacked activity for β-cyano-l-alanine or any of the other nitriles in our set (Supplementary table 1), except for some low activity for the rather large substrate, 3-phenylpropanenitrile (8.5 nKat mg^−1^ enzyme), which neither the wild type nor *At*NIT4 R95T L169A S224Q showed activity for.

## Discussion

NITs are attractive biocatalysts for the synthesis of amides and carboxylic acids for use in the manufacture of drugs and fine chemicals^30^. Novel specificities are generally discovered by environmental sampling^31^ or sequence data mining^32,33^. In some cases, activity against a particular substrate or substrate class has been increased by up to ~3–8-fold by rational design^15^ or directed evolution^34^. We recently saw a ~500-fold improvement in activity for a substrate after a single amino acid exchange and discovered that this correlated with a change in the helical twist of the NIT filament^8^. At the time, the molecular basis for this change was unclear, because we did not have an atomic resolution structure of an active helical NIT. We speculated that the change in substrate size preference resulted from compression of the active-site pocket at the helical interface. This is noteworthy because one of the distinguishing features of branch 1 NITs (EC 3.5.5.1) is that they form helical-, turn-, or spiral-quaternary structures, the basic architecture of these assemblies is conserved in phylogenetically diverse NITs^2–8,35,36^ and appear necessary for activity^21,37^.

Crystal structures of members of the NIT superfamily have been available for almost 20 years^38,39^ and today there >20 crystal structures of various members deposited in the Protein Data Bank (https://www.rcsb.org/). Extensive attempts to crystallize a helical NIT by ourselves and others failed and Thuku et al.^21^ attributed this to the irregular/ ~5-fold symmetry of the NIT helix, which presumably interferes with crystallization. This limits our structural understanding of the active NIT oligomer to ~20 Å electron microscopy information because of the heterogeneous nature of the filaments, until the publication in 2014 by Zhang et al.^15^ of the first helical NIT crystal structure. Interestingly, they observed that 55 C-terminal amino acids were cleaved off before crystallization. This fortuitously led to the formation of filaments with a helical twist of −61° with sufficient flexibility to allow crystallization, but unfortunately also altered the oligomeric structure of the protein and abolished activity (our unpublished results).

Here, we describe the first active NIT filament structure interpretable to atomic resolution. The structure shares conserved features with other members of the superfamily, such as an αßßα core (Figs. 2 and 3a) arranged into dimers across the A-interface^2,21^ and CEEK catalytic tetrad (Fig. 3b)^19,20^. Dimers interact across the C-interface forming left-handed helices with ~5 dimers per turn (Fig. 2). The extended C-terminal tail of each monomer forms two ß-strands, which assemble, along with strands from monomers across the A- and C-interfaces, into a row of 4-stranded ß-sheets in the interior of the tubular filament (Fig. 2). These interactions stabilize the C- and A-interfaces, explaining why C-terminal truncation decreases thermostability^40^ and why the C-terminal tail is an important factor in oligomerization^3,41,42^. Interactions across the helical groove (D- or F-interfaces) were not observed (Fig. 2) and this could explain the plasticity of NIT helical twist^8^.

The architecture of the substrate-binding pocket came as a surprise to us and provided an interesting insight into the structural mechanism of substrate size selection as well as the substrate altering/inducing effect of heterocomplex formation in some plant NITs^8,43^. The NIT superfamily binding pocket has been described^23,24^, but in addition to these shared elements, in *At*NIT4 a loop from the adjacent monomer extends over the C-interface in line with C197 (Fig. 3a, b). We superimposed bound substrates and reaction intermediates from NIT superfamily members^23–25^ on our structure (Supplementary Fig. 2) and propose that this loop places a limit on the maximum bound substrate length (Fig. 3d). Crucially, we observed via molecular dynamics, that this loop is held in position by a charge–charge interaction between R95 and D317. On this basis we refer to the loop as a lid, R95 as a lock, and the flexible ends of the loop as a hinge, which open or close, shifting the lid relative to C197. We previously noted that all ß-cyano-l-alanine converting NIT4s have an arginine in this position, while NIT1s have a histidine and specialist NIT1s have various residues, which correlate with substrate specificity^8^. In addition to this, D137 and surrounding motif: KF**D**FD is conserved in all known NIT4s, while NIT1s have: KL**Y**FD in this position (Supplementary Fig. 1). This may explain why we did not observe any mutations similar to those in NIT1 in our screen, even though many NIT1 substrates were present. This could be tested in the future by mutating residue 317 in addition to the three residues targeted here.

A major challenge in protein engineering is the generation of enzymes with novel substrate specificities. The typical starting point is an enzyme with at least measureable promiscuous activity for the targeted substrate(s)^27^. Rounds of directed evolution or rational engineering are then used to move the protein toward a point of higher activity in the fitness landscape^44^. An analogous process is believed to be responsible for the enormous diversity of modern proteins seen today. Following gene duplication, new enzyme copies, no longer constrained by their original physiological function(s) can diverge by selective pressure on existing promiscuous reactions^45^. However, in nature, broadly specific NIT1 enzymes presumably evolved from a highly specific ancestral NIT4^11^, with no or very low activity for NIT1 substrates. To explore this process we used a focused-library approach, targeting three amino acid residues within the binding pocket that influence substrate specificity disproportionately^27^. We selected altered mutants over one round of directed evolution with a small library of only ~5000 mutants, but which nevertheless sampled at least one of the top ten mutants with 95% probability (Fig. 4). This strategy was effective, generating a mutant (*At*NIT4 R95T L169A S224Q) with altered substrate specificity and no activity against the wild-type substrate.

We observed in our initial screen that some substrates formed a high-absorbance product with the assay reagents, which reduced after incubation with the enzyme, but with no corresponding increase in ammonia (Fig. 5e). We interpret this as evidence of possible amide formation because amide formation accounts for ~50% of the total activity of wild-type *At*NIT4^10^, but we did not explore this phenomenon further. In some cases mutants identified on one substrate showed activity against other substrates when tested using purified enzyme, but showed no growth on these conditions. This phenomenon may result from certain substrates or products being more toxic than others, decomposition of the substrates during incubation or low substrate solubility. In fact this is a key weakness of this strategy and in some cases may mean that interesting variants are lost during selection.

We reconstructed *At*NIT4 R95T L169A S224Q filaments in three dimensions by negative stain EM and found that it has a helical twist of −65°, very similar to *At*NIT3 (Δ*φ* = −66°) (Fig. 6a, c), which has a broad substrate specificity and a preference for large substrates^8^. In contrast to *At*NIT3, the mutant has a larger diameter, more similar to Nit6803 ∆291 (Fig. 6c, d), Nit6803 also shows a very broad substrate specificity^16^. This was fortuitous, because in effect, the two available atomic resolution NIT structures: *At*NIT4 (Δ*φ* = −73°) and Nit6803 ∆291 (Δ*φ* = −60°) represent the helical structure extremes. This allowed us to visualize both structural states, which suggested a mechanism for the observed changes in substrate specificity (Fig. 6e). We propose: R95T eliminates the positive charge at position 95, which disrupts the interaction with D317 and allows the lid loop to open and shift away from C197 (Fig. 3b). In NIT4 R95T this allows 3-phenylpropanenitrile to bind to C197 by opening up the binding pocket, but does not result in a change in helical twist. Introduction of the other two mutations (*At*NIT4 R95T L169A S224Q) results in a large-scale change in helical twist (Fig. 6b, d).

The S224Q exchange on the peripheral border of the binding pocket might have a direct effect on the substrate, as we previously observed for glycine to tryptophan mutants in this position^8^ potentially accounting for the particular combination of substrates observed here. The close proximity of R95 to the catalytic residues may mean that there is direct interaction with or selection for the substrate by a residue in this position. This would account for cases where there is an almost identical helical twist, but mutating this residue still increases activity against a particular substrate^8^. In the case of heterocomplex formation^8,43^, the loop of one subunit and active-site pocket of another subunit interact to produce the observed activity.

These insights have placed us in a position where we can semi-rationally design plant NITs in a very successful way. In fact, the mutant that we generated shows wild-type levels of activity (~300 nKat.mg^−1^ enzyme) for substrates that the wild type is inactive against. This is made possible by the structural flexibility of the lid loop system and explains how by shifting this loop and making a small number of other changes in the binding pocket, a variety of novel substrate specificities evolved in plants from NIT4, a highly specific precursor. More work is needed to further explore this relationship. Certainly the near-atomic resolution structure of a plant NIT1 will allow us to observe the effect of the low-twist helical state on the position of the lid loop. It would also be interesting to target NIT1 sites to determine if the substrate specificity of NIT1 can be further broadened to generate economically interesting enzymes. We also cannot currently explain why *At*NIT4 R95T L169A S224Q shows activity against cyanide, the smallest nitrile (Fig. 5e, f), further work will need to be done to explain this.

## Methods

### NIT expression and purification

His-tagged NITs in pET-21b(+) (Novagen) were expressed at 37 °C in *E. coli* BL21-CodonPlus (DE3)-RIL (Agilent Technologies) for 4 h in LB containing 100 μg ml^−1^ ampicillin and 1 mM IPTG. The cells were pelleted at 4 °C and resuspended in 50 mM Tris–Hcl pH 8.0, 100 mM NaCl with cOmplete^TM^ protease inhibitor (Roche) and sonicated on ice for 4 min (at 15 s intervals) (Sonicator^®^ 3000, Misonix). The clarified supernatant was loaded onto a Ni^2+^-NTA-agarose (Qiagen) column and washed with 50 mM Tris–Hcl pH 8.0, 500 mM NaCl, 20 mM imidazole and eluted over a gradient up to 500 mM imidazole. The NIT-containing peak was pooled and loaded onto a TSKgel PWXL4000 HPLC size exclusion column (Tosoh Corp), equilibrated with 50 mM Tris–Hcl pH 8.0, 100 mM NaCl and the leading edge of the NIT-containing peak was collected. Cryo-EM grids were immediately prepared using this material and aliquots destined for long-term storage were flash frozen in liquid nitrogen and stored at −80 °C after addition of 1 mM DTT. Activity assays were performed after buffer exchange into 50 mM potassium phosphate pH 8.0, 1 mM DTT.

### Negative-stain electron microscopy and image processing

NIT filaments were negatively stained according to standard practices (e.g., Booth et al.^46^). Briefly, 3μl of NIT solution was incubated for 30 s on glow-discharged carbon-coated copper grids before being washed three times with distilled water and stained with two exchanges of 2% uranyl acetate. The grids were loaded into a FEI/Tecnai F20 FEGTEM equipped with a 4k × 4k CCD camera (GATAN US4000 Ultrascan, CA, USA) and imaged at a sampling of 2.11 Å pixel^−1^ at 200 kV with a defocus of −1.0 µm under standard low-dose conditions. Helical segments were extracted using Eman^47^ Boxer with 90% overlap and reconstructed using the iterative helical real-space reconstruction^48^ algorithm using SPIDER^49^ using a featureless cylinder as a starting model as described^8^.

### Cryo-electron microscopy and image processing

Purified *At*NIT4 filaments (2.5 μl at ~0.15 mg ml^−1^) were applied to glow-discharged 1.2/1.3 gold grids (Quantifoil, Micro Tools), incubated for ~30 s, blotted from both sides for ~3.5 s and vitrified using a Vitrobot (FEI) with humidifier turned off (~10% humidity). The grids were imaged on a Titan Krios microscope (FEI) with a K2 Summit direct detector camera (Gatan) using EPU (FEI) at 300 kV. A total of 1266 movies, consisting of 45 frames per movie with a dose rate of 6.5 e^−^ pixel^−1^ s^−1^ over 7 s, were collected over a defocus range of −0.75 to −2.0 µm with a physical pixel size of 0.85 Å pixel^−1^.

### Image processing

RELION^50^ was used for image processing and reconstruction. MotionCor2^51^ was used to correct beam-induced motion and Gctf 1.06^52^ was used to determine parameters for CTF correction. ~3000 particles from straight helical filaments were picked manually from 50 micrographs and used to generate 2D classified averages. The best classes were used as auto-picking templates resulting in ~392,000 particles, poor particles were eliminated by two rounds of 2D classification. Helical reconstruction^53^ was initiated with the known helical parameters of *At*NIT4^8^ using a negative stain reconstruction, which was low-pass filtered to a resolution of 60 Å as a starting model. The best ~133,000 particles were masked, 3D classified, and subjected to 3D autorefinement and postprocessing to yield a map with an overall resolution of 3.4 Å (gold-standard FSC = 0.143 criterion with a soft mask applied to the two half-maps).

### Model building, refinement, and validation

*De novo* polypeptide chain fitting into the map was performed using Buccaneer^54^ within the CCPEM software suite^55^. Obvious errors in connectivity, unbuilt residues, and incorrect rotamers were corrected against the EM map in Coot^56^ and refined using Phenix Real Space Refine^57,58^. After symmetrization in UCSF Chimera^29^ the complete helical assembly was refined using Phenix. Additional rounds of manual fitting and refinement with secondary structure imposed resulted in a final model-to-map correlation of 0.8.

### Data visualization

All molecular visualization and high quality image rendering were performed using UCSF Chimera^29^.

### Molecular dynamics

The molecular dynamics simulation tool using default parameters was used in UCSF Chimera^29^.

### Substrate correlation value

Plant NITs for which substrate specificity data is available were used: *A. thaliana* NIT1, NIT3^12^, and NIT4^10^ (*At*NIT1: NP_851011.1; *At*NIT3: NP_190018.1; *At*NIT4: NP_197622.1); *Salix alba* NIT1-3^14^ (*Sal*NIT1-3: MH567030); *Capsella rubella* NIT1 and NIT2^8^ (*Cr*NIT1: XP_006291436.1; *Cr*NIT2: XM_006284056.1). Sequences were allocated to NIT4, NIT1, and specialist NIT1 groups and compared in pairs: amino acid positions were allocated one point when a residue differed between the groups and the substrate differed, or when the residue remained the same when the substrate remained the same. Points were subtracted when this was not the case.

### Library construction

The *A. thaliana* nitrilase 4 (*AT5G22300*) pET-21b(+) vector was a gift from Markus Piotrowski (Ruhr-Universität Bochum). Sequential randomization of the chosen sites was achieved with NNS (N = A/T/C/G; S = G/C) degenerate codons applying a modified QuikChange^®^ PCR protocol^59^ using HiFi Hotstart Readymix (Kappa Biosystems). PCR reactions (50 μl) with 1–2 ng of template DNA were run under the following conditions: 95 °C for 3 min (1 cycle); 98 °C for 20 s, 62 °C for 15 s, 72 °C for 4 min (20–30 cycles); and 72 °C for 10 min (1 cycle) and subsequently digested with DpnI at 37 °C for 1 h. Primers are listed in Supplementary Table 2. Sufficient DNA for large-scale transformation was obtained by pooling several rounds of PCR product and gel purifying (Zyppy™ Gel Purification Kit, Zymo Research). Transformations were completed by electroporation in 400 μl electroporation cuvettes (Eppendorf). DNA sequencing was performed by the Central DNA Sequencing Facility (University of Stellenbosch, South Africa).

### Statistics and reproducibility

The theoretical library size required for site-saturation mutagenesis on three amino acid residues was estimated using the TopLib calculator^26^ (http://stat.haifa.ac.il/~yuval/toplib/); 100% yield was assumed. A sample consisting of 1% of the volume of the recovered cells was plated out to estimate the total number of cells transformed. The resulting colonies were counted and a confidence interval was derived using: (*n* ± z*√*n*) * *N*, where *n* is the number of colonies counted, z* are the values from a standard normal distribution for the appropriate probability level, and *N* is the sampling^60^. Oversampling requirements were calculated using the formula: P(Missing 0 mutants) = e^−λ^ where λ = *n*(1 – 1·*n*^*−1*^)^*m*^ for *n* unique mutants in a library of size *m*^61^. Activity assays and selection experiments were performed at least in triplicate; quartiles, medians and standard deviations were calculated in Microsoft Excel.

### Selection

Minimal media agar plates without nitrogen were made according to an adapted protocol^62^. M9 minimal salts with agar (bacteriological agar, Merck) was supplemented with 0.4% glucose, 0.1 mM CaCl_2_, 2 mM MgSO_4_, 1 mM IPTG, and ampicillin (100 μg ml^−1^) and one of a set of 42 nitriles (10 mM) (Supplementary Table 1) added to the warm agar. Nitrile stocks (250 mM) were made up by vortexing in distilled water at 37 °C where possible. Hydrophobic nitriles were dissolved in a gradually increasing volume of methanol at 37 °C while vortexing and made up to the correct volume with warm water. In some cases with highly water insoluble nitriles small particles remained after vortexing in 100% methanol, these were used as is. Library plasmid DNA was transformed into BL21 *E. coli* according to calculated oversampling requirements. The cells were pelleted and washed with ice-cold 1× minimal salts to remove any source of nitrogen; ~4 × 10^5^ transformants were plated on each nitrile plate. *E. coli* transformed with *At*NIT4 was grown on selection plates containing β-CN(Ala) as a positive control, while *At*NIT4 was grown on 4-hydroxyphenyacetonitrile, 6-heptenenitrile, mandelonitrile, butanenitrile, 4-cyanopyridine, fluoroacetonitrile, fumaronitrile, potassium cyanide, 2-furonitrile, and 2-cyanopyridine as negative controls. Untransformed *E. coli* on plates lacking nitriles did not form observable colonies. The plates were grown over several days at 37 °C.

### Activity assays

Specific activity measurements were conducted using the indophenol blue assay^43^ or Nessler reagent assay^63^ with substrates purchased from Sigma-Aldrich. Briefly, 2.5 mM substrate, 1 mM DTT and between 0.5 and 10 μg of protein were incubated in 100 μl reaction tubes containing phosphate buffer at 37 °C in intervals of between 5 and 30 min. Initial substrate screening was performed as above, except that 2 μg of protein and 10 mM substrate were incubated for 2 h. Nessler assay: 100 μl of 100 mM KI, 27 mM HgCl_2_ and 330 mM NaOH was added immediately following incubation and left at room temperature for 10 min before measuring the absorbance at 420 nm. Indophenol blue assay: reactions were terminated by the addition of 4 μl of 100 mM phenol (in ethanol), followed by the addition of 4 μl of 19 mM sodium nitroprusside and 10 μl of oxidizing solution (1 part of 470 mM NaClO and 4 parts of 775 mM Na_3_C_6_H_5_O_7_ and 256 mM NaOH) in distilled water and boiled for 4 min, 1 ml distilled water was added before measuring absorbance at 640 nm. These procedures were conducted in triplicate. Controls consisted of identical reaction mixtures containing enzyme inactivated by boiling for 5 min prior to addition.

### Reporting summary

Further information on research design is available in the Nature Research Reporting Summary linked to this article.

## Supplementary information

Reporting Summary

Supplementary Information

## Data Availability

Expression plasmids generated in this study are available from the corresponding author upon reasonable request. Supplementary Tables 1–5 summarize the data underlying Figs. 3–5. The NIT maps and coordinates have been deposited in the Electron Microscopy Data Bank (http://www.ebi.ac.uk/pdbe/emdb/) and Protein Data Bank (https://www.rcsb.org/): *At*NIT4 (Δ*ϕ* = −73.0°) EMD-0320, pdb id: 6i00; *At*NIT4 R95T L169A S224Q EMD-4406; *At*NIT4 R95T (Δ*ϕ* = −73.3°) EMD-4804, Nit6803 Δ291 EMD-4407. Protein sequences are available from Genbank: *At*NIT1 NP_851011.1; *At*NIT3 NP_190018.1; *At*NIT4 NP_197622.1; *Cr*NIT1 XP_006291436.1; *Cr*NIT2 XP_006284056.1; *Sal*NIT1-3 MH567030; Nit6803 BAA10717.1.
