# Supplementary material for: Cryo-EM and directed evolution reveal how Arabidopsis nitrilase specificity is influenced by its quaternary structure
Source: Commun Biol. 2019 Jul 17;2:260. doi: 10.1038/s42003-019-0505-4 (PMC6637149; doi:10.1038/s42003-019-0505-4)
Supplement: Supplementary file 2 — Supplementary Information [file 42003_2019_505_MOESM2_ESM.pdf]

Supplementary Figures

```
AtNIT4      1 MSMQQETSHMTAAPQTNGHQIFPEIDMSAGDSSSIVRATVVQASTVFYDTPATLDKAERLLSEAAENGSQ
AtNIT1      1 -----------MSSTKDMSTVQNATPFNGVAPSTTVRVTIVQSSTVYNDTPATIDKAEKYIVEAASKGAE
CrNIT2      1 ---------------MSGTEQIMSTKFNGVASPTTVRATIVQASTVYNDTPATLEKAKKYIEEAASNGSE
CrNIT1      1 ---------------MSSTVKNTADPVDGVAPSSIVRVTIVQASTVYNNTPATLDKAEKYVVEAASKGAK
SalNIT1-3   1 ---------------MSKTTLKDTTPVSNDFPSTIVRATIVQASSVYNDTPKTLEKAEKFIAEAASDGAQ
AtNIT3      1 -----------MSSTEEMSSVKNTTQVIGVDPSSTVRVTIVQSSTVYNDTPATLDKAEKFIVEAASKGAK
Nit6803    -2 --------------------GSHMLGKIMLNYTKNIRAAAAQISPVLFSQQGTMEKVLDAIANAAKKGVE
                                                     β1                     α1

AtNIT4     71 LVVFPEAFIGGYPRGSTFELAIGSRTAKGRDDFRKYHASAIDVPGPEVERLALMAKKYKVYLVMGVIERE
AtNIT1     60 LVLFPEGFIGGYPRGFRFGLAVGVHNEEGRDEFRKYHASAIHVPGPEVARLADVARKNHVYLVMGAIEKE
CrNIT2     56 LVVFPEAFIGGYPRGFRFGLAVGVFNEEGRDEFRKYHASAIPVPGPEVEKLADMAGKNNVYLVMGAIEKD
CrNIT1     56 LVLFPEAFVGGYPRGFRFGLATGVHNEEGRDEFRKYHASAIKVPGPEVERLAELAGKTNVYLVMGAIEKD
SalNIT1-3  56 LVVFPEAYVGGYPRGFRFGMAVGVQNEEGRDLFRKYHASAIAVPGPEVDKLAEMARKYKVYLVTGAIEKD
AtNIT3     60 LVLFPEAFIGGYPRGFRFGLAVGVHNEEGRDEFRNYHASAIKVPGPEVERLAELAGKNNVHLVMGAIEKD
Nit6803    48 LIVFPETFVPYYPYFSFVEPP----VLMGKSHLKLY-QEAVTVPGKVTQAIAQAAKTHGMVVVLGVNERE
              β2          α2                 α2*                  α3          β3

AtNIT4    141 GYTLYCTVLFFDSQGLFLGKHRKLMPTALERCIWGFGDGSTIPVFDTPIGKIGAAICWENRMPSLRTAMY
AtNIT1    130 GYTLYCTVLFFSPQGQFLGKHRKLMPTSLERCIWGQGDGSTIPVYDTPIGKLGAAICWENRMPLYRTALY
CrNIT2    126 GYTLYCTALFFSPQGQFLGKHRKLMPTTLERCIWGQGDGSTIPVYDTPIGKLGAAICWENRMPLYRTALY
CrNIT1    126 GYTLYCTALFFSPQGQFLGKHRKLMPTTLERCIWGQGDGSTIPVYDTPIGKLGAAICWENRMPLYRTALY
SalNIT1-3 126 GYTLYCIALFFSSEGHFLGKHRKVMPTAMERVIWGYGDGSTLPVYDTPLGKLGGAICWENRMPSLRTSLY
AtNIT3    130 GYTLYCTALFFSPQGQFLGKHRKVMPTSLERCIWGQGDGSTIPVYDTPIGKIGAAICWENRMPLYRTALY
Nit6803   113 EGSLYNTQLIFDADGALVLKRRKITPTYHERMVWGQGDGAGLRTVDTTVGRLGALACWEHYNPLARYALM
                  β4           β5         α4              β6      β7          α5

AtNIT4    211 AKGIEIYCAPTADS------RETWLASMTHIALEGGCFVLSANQFCRRKDYPSPPEYMFSGSEESLTPDS
AtNIT1    200 AKGIELYCAPTADG------SKEWQSSMLHIAIEGGCFVLSACQFCQRKHFPDHPDYLFTDWYDDKEHDS
CrNIT2    196 AKGIEIYCAPTADG------SKEWQSSMLHIAIEGGCFVLSACQFCQRKDFPEHPDYLFTDLDENKEQDA
CrNIT1    196 AKGIEIYCAPTADG------SKEWQSSMMHIALEGGCFVLSACQFCLRKDFPDHPDYLFTDWEDYKEDDA
SalNIT1-3 196 AKGVELYCAPTADW------SKEWQSSVMHIAMEGGCFVMAACQFCLRKDYPEDPNYYFTDESDDHSPEA
AtNIT3    200 AKGIEIYCAPTADY------SLEWQASMIHIAVEGGCFVLSAHQFCKRREFPEHPDYLFNDIVDTKEHDP
Nit6803   183 AQHEQIHCGQFPGSMVGQIFADQMEVTMRHHALESGCFVINATGWLTAEQK-------LQITTDEKMHQA
                     β8               α6               β9           α6*

AtNIT4    275 VVCAGGSSIISPLGIVLAGPNYRGEALITADLDLGDIARAKFDFDVVGHYSRPEVFSLNIREHPRKAVSF
AtNIT1    264 IVSQGGSVIISPLGQVLAGPNFESEGLVTADIDLGDIARAKLYFDSVGHYSRPDVLHLTVNEHPRKSVTF
CrNIT2    260 IVSQGGSVIISPLGQVLAGPNFESEGLITADLDLGEIARAKLYFDVVGHYTKPDVLSLTVNEDPKKTVTF
CrNIT1    260 IVSQGGSVIISPLGQVLAGPNFESEGLITADLDLGDVARAKLYFDAVGHYSRPDVLHLTVNEHPKKTVTF
SalNIT1-3 260 VVSPGGSVIISPLGKILAGPNFGSEGLVTADLDLGDIAKAKLYFDVVGHYSRPDIFNLTVNEKEKKPVTF
AtNIT3    264 TVSGGGSVIISPLGKVLAGPNYESEGLVTADLDLGDIARAKLYFDVVGHYSKPDIFNLTVNEHPKKPVTF
Nit6803   246 LSGGCYTAIISPEGKHLCEPIAEGEGLAIADLDFSLIAKRKRMMDSVGHYARPDLLQLTLNNQPWSALEA
                     β12      β13         β14        α7                   β15      β16

AtNIT4    345 KTSKVMEDESV--------------------
AtNIT1    334 VTKVEKAEDDSNK------------------
CrNIT2    330 VSKVEKAEDASNK------------------
CrNIT1    330 MTKVEKAEDDSNK------------------
SalNIT1-3 330 VSKPEDDSEPQDK------------------
AtNIT3    334 MTKVEKAEDESNK------------------
Nit6803   316 NPVTPNAIPAVSDPELTETIEALPNNPIFSH
```

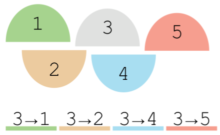

α-helix
β-strand
Catalytic residues
Targeted residues
Binding pocket
Not visualized

Supplementary Figure 1. Structural sequence alignment[1] between NIT's relevant for this work. β-strands and α-helices are colored purple and yellow, respectively and numbered according to Thuku et al[2]. Residues making up the catalytic tetrad are shown in magenta and the residues targeted here for site-saturation mutagenesis are shown in cyan. Regions forming the borders of the substrate-binding pocket are shown in gray. Helical interfaces are shown in light green, tan, light blue and orange and explained in the inset, residues not visualized in the model are outlined in red. *At*NIT1, *At*NIT3, *At*NIT4: nitrilase 1, 3 and 4 from *Arabidopsis thaliana*; *Cr*NIT1, *Cr*NIT2: nitrilase 1 and 2 from *Capsella rubella*; *Sal*NIT1-3: nitrilase 1-3 from *Sinapis alba*; Nit6803: nitrilase from *Synechocystis sp*. PCC6803 (3wuy[3]).

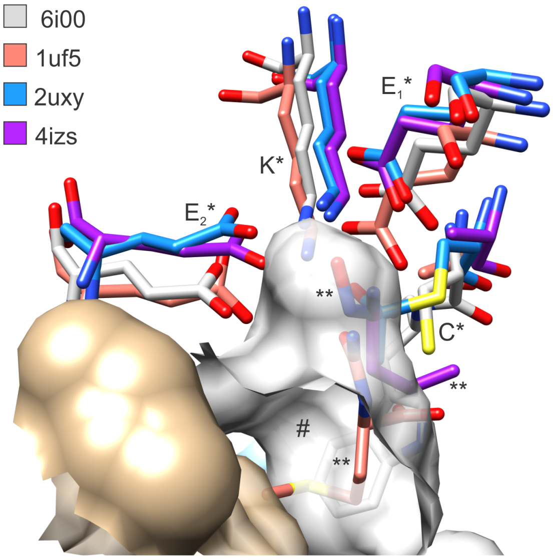

Supplementary Figure 2. NIT4 and nitrilase superfamily members with bound substrates/ intermediates. The positions of the CEEK catalytic tetrad residues (*) are conserved between *At*NIT4 and other nitrilase superfamily members after structural alignment. Substrates/ substrate intermediates (**) localize to the edge of the *At*NIT4 binding pocket (gray surface, #) and extend towards the lid loop (tan). The structures used were: a C171A/V236A mutant of N-carbamyl-D-amino acid amidohydrolase from *Agrobacterium sp.* KNK712 complexed with N-carbamyl-D-methionine (pdb id: 1uf5)[4]; an amidase from *Pseudomonas aeruginosa* with trapped acyl transfer intermediate (pdb id: 2uxy)[5] and a C145A mutant of the amidase from *Nesterenkonia* AN1 complexed with butyramide substrate (pdb id: 4izs)[6].

Supplementary Tables

Supplementary Table 1. Substrate correlation scores.

	Score: ∑(correlated)- ∑(uncorrelated)					
	**P_{166}**	**T_{167}**	**A_{168}**	**L_{169}**	**E_{170}**	**R_{171}**
**D_{223}**	2	2	1	5	2	2
**S_{224}**	7	7	6	**10**	7	7
**R_{225}**	5	5	4	8	5	5
**E_{226}**	3	3	2	6	3	3
**T_{227}**	3	3	2	6	3	3

Supplementary Table 2. Primer sequences

**Primer**		**T_m**
R95NNS_Forward	GGT TCT **NNS** ACC GCT AAA GCA CGA GAT GAC	68.6° -
R95NNS_Reverse	GC GGT **SNN** AGA ACC AAT AGC CAA TTC AAA GG	78.9°
L169NNS_Forward	CA GCT **NNS** GAA CGT TGC ATT TGG GGA TTT GG	69.1° -
L169NNS_Reverse	CG TTC **SNN** AGC TGT AGG CAT GAG TTT GCG	77.7°
S224NNS_Forward	CT GCT GAT **NNS** AGA GAA ACT TGG CTA GCA TCA ATG ACT CAT ATT GCA CTT GAG	80.1° -
S224NNS_Reverse	GC TAG CCA AGT TTC TCT **SNN** ATC AGC AGT AGG TGC ACA ATA AAT CTC AAT GCC	82.4°
R95T_Forward	GGT TCT **ACT** ACC GCT AAA GCA CGA GAT GAC	71.8°
R95T_Reverse	GC GGT **AGT** AGA ACC AAT AGC CAA TTC AAA GG	72.8°

Supplementary Table 3. Library assembly bottlenecks. The number of CFU's estimated from a 1% sample and the number required at the 95% confidence level.

	**# CFU's**			
	**Counted**[7]	**Full coverage**[8]	**Top mutant**[9]	**In top ten**[9]
**1 mutation**	1610 ± 790	172	80	7
**2 mutations**	3400 ± 1140	8130	2130	138
**3 mutations**	5200 ± 1410	3.42×10^5	55500	2970
**DH5α**	4.08×10^6	$(6.02\pm1.77)\times10^4$	-	-
**BL21**	7.71×10^5	$(6.02\pm1.77)\times10^4$	-	-
**# Plated out**	4×10^5	-	-	-

Supplementary Table 4. Nitriles included in the selection assay. All of the nitriles were supplied by Sigma-Aldrich with % purity shown. Those nitrile plates that produced bacterial colonies are shown followed by brackets indicating the number of colonies observed on the plate and the number of days required for visible colony growth.

**Nitrile (purity, # colonies, # Days)**	
Saturated aliphatic mononitriles	***Benzene derivatives***
Potassium Cyanide ≥98% (1, 9)	Benzonitrile 99.9%
Acetonitrile 97%	Phenylacetonitrile 98%
Propanenitrile 99% (7, 2)	3-Phenylpropionitrile 99%
Butanenitrile ≥99% (92, 2)	4-phenylbutyronitrile 99%
Pentanenitrile 99.5%	Mandelonitrile 97%
Octanenitrile 97%	3-Hydroxy-3-phenylpropionitrile ?%
Nonanenitrile 98%	2-Amino-2-phenylacetonitrile 98%
Dodecanenitrile 99% (150, 13)	***Aryl nitriles***
Fluoroacetonitrile 98% (300, 2)	4-hydroxyphenylacetonitrile 98%
Unsaturated aliphatic mononitriles	3- Cyanophenol 99%
Acrylonitrile 99.5% (20, 9)	p-Tolunitrile 98%
3-Butenenitrile 98% (43, 13)	o-Tolunitrile ≥97% (1, 13)
4-Pentenenitrile 97% (4, 9)	m-Tolunitrile 99%
6-Heptenenitrile 98% (5, 9)	2-Nitrobenzonitrile ≥99%
Branched aliphatic mononitriles	3-Nitrobenzonitrile 98%
Isovaleronitrile 98% (3, 4)	3-Aminobenzonitrile 99%
ß-cyano-L-alanine 95%	3,5 Dibromo-4-hydroxybenzonitrile (Bromoxynil) 99.6%
Saturated aliphatic dinitriles	3-Chlorobenzonitrile 99%
Malononitrile ≥99%	4-Chlorobenzonitrile 99%
Adiponitrile 99% (22, 2)	***Unsaturated aliphatic dinitriles***
Heterocyclic compounds	Crotonitrile 99%
2 – Furonitrile 99%	Fumaronitrile 98%
2-Cyanopyridine 99% (1, 9)	***Aromatic dinitriles***
3-Cyanopyridine 98% (25, 13)	Benzylidenemalononitrile 98%
4 Cyanopyridine 98% (300, 2)	1,4-dicyanobenzene 98%

Supplementary Table 5. Substrate profiles of *At*NIT4 and *At*NIT4 R95T L169A S224Q. The data show means ± standard deviation (n = 3) n.d.: not detectable

	**Specific enzyme activity [nkat.(mg protein)$^{-1}$]**	
**Substrate**	***At*NIT4**	***At*NIT4 R95T L169A S224Q**
Fumaronitrile	n.d.	n.d.
4-cyanopyridine	n.d.	27.24 ± 1.01
Fluoroacetonitrile	n.d.	34.31 ± 4.88
2-furonitrile	n.d.	232.89 ± 6.82
2-cyanopyridine	n.d.	340.58 ± 18.63
Potassium cyanide	n.d.	378.56 ± 19.37
ß-cyano-L-alanine	314.16 ± 71.52	n.d.

Supplementary references

1. Meng, E. C., Pettersen, E. F., Couch, G. S., Huang, C. C. & Ferrin, T. E. Tools for integrated sequence-structure analysis with UCSF Chimera. *BMC Bioinformatics* **7**, 1–10 (2006).

2. Thuku, R. N., Brady, D., Benedik, M. J. & Sewell, B. T. Microbial nitrilases: versatile, spiral forming, industrial enzymes. *J. Appl. Microbiol.* **106**, 703–27 (2009).

3. Zhang, L. *et al.* Structural insights into enzymatic activity and substrate specificity determination by a single amino acid in nitrilase from Syechocystis sp. PCC6803. *J. Struct. Biol.* **188**, 93–101 (2014).

4. Hashimoto, H. *et al.* Crystal Structure of C171A/V236A mutant of N-carbamyl-D-amino acid amidohydrolase. *RCSB Protein Data Bank* (2003). doi:10.2210/pdb1uf5/pdb

5. Andrade, J., Karmali, A., Carrondo, M. a & Frazão, C. Structure of amidase from Pseudomonas aeruginosa showing a trapped acyl transfer reaction intermediate state. *J. Biol. Chem.* **282**, 19598–605 (2007).

6. Kimani, S. W., Hunter, R., Vlok, M., Watermeyer, J. & Sewell, B. T. Covalent modifications of the active site cysteine occur as a result of mutating the glutamate of the catalytic triad in the amidase from Nesterenkonia sp. *RCSB Protein Data Bank* (2013). doi:10.2210/pdb4izs/pdb

7. Abbott, J. H. & Rosenblatt, J. I. Two stage estimation with one observation on the first stage. *Ann. Inst. Stat. Math.* **14**, 229–235 (1962).

8. Denault, M. & Pelletier, J. N. Protein Library Design and Screening BT - Protein Engineering Protocols. in (eds. Arndt, K. M. & Müller, K. M.) 127–154 (Humana Press, 2007). doi:10.1385/1-59745-187-8:127

9. Nov, Y. When second best is good enough: Another probabilistic look at saturation mutagenesis. *Appl. Environ. Microbiol.* **78**, 258–262 (2012).
